# Enhancing effect of lysine combined with other compounds on cephamycin C production in *Streptomyces clavuligerus*

**DOI:** 10.1186/1471-2180-13-296

**Published:** 2013-12-20

**Authors:** Carla A Leite, André P Cavallieri, Maria L G C Araujo

**Affiliations:** 1 Department of Biochemistry and Technological Chemistry, UNESP - São Paulo State University, Institute of Chemistry, 14800-900 Araraquara, SP, Brazil

**Keywords:** *Streptomyces clavuligerus*, Cephamycin C, Lysine, Diamines, Alpha-aminoadipic acid, Response surface

## Abstract

**Background:**

Lysine plays an important role in *Streptomyces clavuligerus* metabolism; it takes part in its catabolism, via cadaverine, and in its secondary metabolism, in which lysine is converted via 1-piperideine-6-carboxylate to alpha-aminoadipic acid, a beta-lactam antibiotic precursor. The role of lysine as an enhancer of cephamycin C production, when added to production medium at concentrations above 50 mmol l^-1^, has already been reported in the literature, with some studies attributing a positive influence to multifunctional diamines, among other compounds. However, there is a lack of research on the combined effect of these compounds on antibiotic production.

**Results:**

Results from experimental design-based tests were used to conduct response surface-based optimization studies in order to investigate the synergistic effect of combining lysine with cadaverine, putrescine, 1,3-diaminopropane, or alpha-aminoadipic acid on cephamycin C volumetric production. Lysine combined with cadaverine influenced production positively, but only at low lysine concentrations. On the whole, higher putrescine concentrations (0.4 g l^-1^) affected negatively cephamycin C volumetric production. In comparison to culture media containing only lysine as additive, combinations of this amino acid with alpha-aminoadipic acid or 1,3-diaminopropane increased cephamycin C production by more than 100%.

**Conclusion:**

This study demonstrated that different combinations of lysine with diamines or lysine with alpha-aminoadipic acid engender significant differences with respect to antibiotic volumetric production, with emphasis on the benefits observed for lysine combined with alpha-aminoadipic acid or 1,3-diaminopropane. This increase is explained by mathematical models and demonstrated by means of bioreactor cultivations. Moreover, it is consistent with the positive influence of these compounds on lysine conversion to alpha-aminoadipic acid, a limiting step in cephamycin C production.

## Background

The commercial importance of the actinomycete *Streptomyces clavuligerus* lies in its ability to produce several secondary metabolites of therapeutic interest [1]. Among these compounds are: cephamycin C, a beta-lactam antibiotic more resistant to beta-lactamases than the structurally similar antibiotic cephalosporin C produced by filamentous fungi, and for this reason used as raw material for production of semi-synthetic antibiotics (cefotetan, cefoxitin, cefmetazole, and temocillin) [2,3]; clavulanic acid, a beta-lactamases inhibitor whose use in conjunction with amoxicillin is the most important commercial example [4]; other clavams, which have antifungal properties [5]; and non-beta-lactam compounds such as holomycin and tunicamycin, which have antibiotic and antitumor properties [5-7]. The biosynthetic diversity inherent to *S. clavuligerus* results in extremely complex metabolic regulation [8-14], which has led to different studies aimed at increasing the biosynthesis of relevant biocompounds. Among these compounds, cephamycin C has been one of the most extensively investigated [15-23]. The basic structure of this biocompound and of all other beta-lactam antibiotics produced by prokaryotes or eukaryotes derives from L-cysteine, L-valine, and L-alpha-aminoadipic acid. In prokaryotes, alpha-aminoadipic acid is the product of lysine degradation via 1-piperideine-6-carboxylate [24-26]. The use of exogenous lysine to enhance cephamycin C biosynthesis in cultures of producer species has been known for over thirty years [16,20,23,27,28]. Studies have shown that high lysine concentrations (above 50 mmol l^-1^) promote higher cephamycin C production as compared to that of culture media containing little or no lysine. Researchers have obtained increases of up to 300% in cephamycin C production by *S. clavuligerus* in a culture medium containing about 100 mmol l^-1^ of lysine [14,20,21]. In spite of lysine degradation via 1-piperideine-6-carboxylate pathway producing the precursor alpha-aminoadipic acid [25,26], complete lysine catabolism occurs via cadaverine [24,29,30]. Cadaverine and other diamines, such as diaminopropane and putrescine, promote beta-lactam antibiotic production in *Nocardia lactamdurans* or *S. clavuligerus *[31-34]. Nevertheless, it is difficult to determine the extent to which these compounds influence antibiotic biosynthesis, since diamines act as modulators of several cell functions [32,33,35]. Thus, there is scarce quantitative research on the use of lysine combined with other diamines or other compounds that can potentially enhance beta-lactam antibiotic production in *S. clavuligerus *[16,23,33]. This was explored in this study, which investigates increases in cephamycin C production by adding cadaverine, putrescine, 1,3-diaminopropane or alpha-aminoadipic acid in culture media containing lysine as compared to those obtained in culture media containing lysine alone. Cultivations were performed in accordance with a central composite-based, face-centered experimental design (CCF) whereas concentrations of lysine combined with every compound were optimized using Response Surface Methodology. Best conditions were validated by means of batch cultivations in a stirred and aerated bench-scale bioreactor.

## Methods

### Microorganisms

*Streptomyces clavuligerus* ATCC 27064 was stored in the form of spore suspension (approximately 10^8^ spores ml^-1^) at -80°C in 2 ml cryotube vials (glycerol at 20% w v^-1^).

*Escherichia coli* ESS 2235 supersensitive to beta-lactam antibiotics was employed as test organism. The strain was cultivated in nutrient agar medium (Difco™ Nutrient Agar) at 37°C for 24 hours. The cells were stored at -80°C in 2 ml cryotube vials.

### Culture media

The seed medium contained (g l^-1^) tryptone (5.0), yeast extract (3.0), malt extract (10), and buffering agent 3-(N-morpholine) propanesulfonic acid (MOPS) (21). The inoculum medium consisted (g l^-1^) of soluble starch (10), cotton seed extract (PROFLO® - Traders Protein, USA) (8.5), yeast extract (1.0), K_2_HPO_4_ (0.80), MgSO_4_.7H_2_O (0.75), MOPS (21), and 10 ml of salt solution per l of medium. The salt solution contained (g l^-1^) MnCl_2_.4H_2_O (1.0), FeSO_4_.7H_2_O (1.0), and ZnSO_4_.7H_2_O (1.0). The basal production medium contained (g l^-1^) soluble starch (10), PROFLO® (8.5) boiled down and filtered (using a vacuum pump), yeast extract (0.50), K_2_HPO_4_ (1.75), MgSO_4_.7H_2_O (0.75), CaCl (0.20), NaCl (2.0), MOPS (21), the aforementioned salt solution (5.0 ml l^-1^), and sodium thiosulfate (1.0) added at 30 h after inoculation according to Inamine and Birnbaum [31]. The initial pH of culture media was fitted to 6.8 ± 0.1. The proportion of filtered PROFLO® nitrogen corresponded to 40% of gross PROFLO®.

In order to investigate the influence of different compounds on cephamycin C biosynthesis, the production medium was supplemented with lysine, alpha-aminoadipic acid, and diamines 1,3-diaminopropane, putrescine, and cadaverine. These compound concentrations were established according to the purpose of each experiment.

### Experimental procedure

Spore germination and inoculum preparation consisted of two pre-cultures with 24-hour cultivation each in shake flasks. Inoculum volume comprised 10% of suspension cell volume per culture medium volume throughout this study. Submerged cultures for cephamycin C production were performed in 500 ml Erlenmeyer shake flasks at 28°C and 260 rpm (5 cm eccentricity). To prevent problems of oxygen limitation during the shake-flask procedure, the broth volume was kept under 10% of the Erlenmeyer flask nominal volume. Samples were collected at 24-hour intervals. Experiments in the bench-scale bioreactor (New Brunswick Bioflo 2000; 5 l working volume) were performed at 1.0 vvm aeration rate, 6.8 ± 0.1 pH, 28°C temperature, and 50% dissolved oxygen saturation level automatically controlled by varying the agitation speed.

### Analytical methods

The supernatant was obtained after centrifugation of the culture medium at 15,550 x g for 10 min, 4°C, for further analyses. The cell density was quantified as grams of dry weight per liter of sample (gDWC l^-1^). Cephamycin C was determined by means of the agar-diffusion assay method using cephalosporin C zinc salt (Sigma) as standard. Penase® (BD Difco) was employed at 20 μL per ml of sample, reacting at 25°C for 20 min to degrade penicillin N. In this method, the measure of cephamycin C represents the total amount of cephalosporins in the sample (in mg l^-1^) [36]. A calibration curve was performed using ten cephalosporin C concentration values from 5 to 120 mg l^-1^ and 24 replicates for each concentration. Antibiotic analyses were also carried out via high-performance liquid chromatography as described in Baptista Neto et al. [37]. Lysine and alpha-aminoadipic acid analyses were conducted by means of the post-column derivatization method with orthophtalaldehyde and quantified in a fluorescence detector [38]. The starch concentration was determined after acid hydrolysis, by quantifying the total reducing sugars by the dinitrosalicylic acid method [39].

### Experimental design

CCF experimental designs, including four replicates of an experiment under the same conditions, were employed to investigate individual and combined effects of lysine and compounds, one at a time, putrescine, 1,3-diaminopropane, cadaverine, and alpha-aminoadipic acid, on cephamycin C production. The response surface methodology was used to investigate the relationship between cephamycin C production (response variable) and the compounds that enhance beta-lactam antibiotic production (independent variables) [40,41]. The chosen experimental design and the established concentration limits of the compounds under investigation (independent variables) were appropriate for adjusting models represented by second-order polynomials according to the following equation:

$$\hat{y}=a_{0}+a_{1}x_{\mathrm{Lys}}+a_{i}x_{i}+a_{11}x_{\mathrm{Lys}}^{2}+a_{\mathrm{ii}}x_{i}^{2}+a_{1_{i}}x_{\mathrm{Lys}}x_{i}$$

where $\hat{y}$ represents the dependent variable, cephamycin C production (mg l^-1^), a_0_ is the interception coefficient (average value of cephamycin C, in mg l^-1^), x represents the independent variables in coded unities: *x*_*Lys*_ = lysine, and *x*_*i*_ represents the other compounds studied (i = alpha-aminoadipic, 1,3-diaminopropane, cadaverine or putrescine); a_1_ and a_i_ are the linear terms, a_11_ and a_ii_ are the quadratic terms, a_1i_ is the interaction term. *Statistica* software (7.0 version) was used for regression and graphical analyses of experimental data.

The conditions of independent variables and cephamycin C production results (observed and predicted) are shown in Tables 1 and 2.

**Table 1:** Range and levels of the independent variables lysine (Lys) and alpha-aminoadipic acid (AAA), in coded and original units, according to the two-factor, three-level central-composite-based, face-centered, experimental design (CCF); the response variable is cephamycin C concentration (CephC) obtained at 72-hour cultivation

**Run**	**Independent variables**				**Response**	
	**Coded units**		**Original units (g l**^**-1**^**)**		**CephC (mg l**^**-1**^**)**	
	***x***_***Lys***_	***x***_***AAA***_	***x***_***Lys***_	***x***_***AAA***_	Measured*	**Predicted**
1	-1	-1	0.9	0	25.0 ± 8.2	15.5
2	0	-1	3.2	0	45.0 ± 9.6	52.7
3	+1	-1	5.5	0	55.0 ± 5.9	56.7
4	-1	0	0.9	0.32	44.1 ± 0.9	57.8
5	0	0	3.2	0.32	105.8 ± 6.6	100.5
6	+1	0	5.5	0.32	118.5 ± 6.4	110.0
7	0	+1	3.2	0.64	112.4 ± 0.0	110.6
8	0	+1	3.2	0.64	102.8 ± 0.0	110.6
9	0	+1	3.2	0.64	117.8 ± 0.0	110.6
10	0	+1	3.2	0.64	112.0 ± 0.0	110.6
11	-1	+1	0.9	0.64	66.7 ± 7.7	62.4
12	+1	+1	5.5	0.64	118.8 ± 9.6	125.6

*The cultivations were performed in triplicate, with the exception of cultivation at condition (0,+1) performed in quadruplicate; SD = standard deviation.

**Table 2:** Range and levels of independent variables lysine (Lys), 1,3-diaminopropane (1,3D), cadaverine (Cad), and putrescine (Put), in coded and original units, according to two-factor, three-level central-composite-based, face-centered, experimental designs (CCF); the response variable is cephamycin C concentration (CephC) obtained at 72-hour cultivation

	**Independent variables**						**Response**					
	**Coded units**		**Original units (g l**^**-1**^**)**				**CephC (mg l**^**-1**^**)**					
							**Lys + 1,3D**		**Lys + Cad**		**Lys + Put**	
**Run**	***x***_***Lys***_	***x***_***i***_	***x***_***Lys***_	***x***_***1,3D***_	***x***_***Cad***_	***x***_***Put***_	Measured*	**Predicted**	Measured*	**Predicted**	Measured*	**Predicted**
1	-1	-1	0.0	0.0	0.0	0.0	18.1 ± 3.0	10.6	19.0 ± 2.7	22.7	18.0 ± 2.7	16.7
2	0	-1	3.7	0.0	0.0	0.0	45.6 ± 7.2	59.9	45.6 ± 2.2	39.1	47.3 ± 3.2	53.9
3	+1	-1	7.4	0.0	0.0	0.0	72.3 ± 4.1	64.9	72.1 ± 1.9	74.7	75.5 ± 3.6	70.3
4	-1	0	0	2.5	3.5	0.2	47.6 ± 3.9	53.9	34.7 ± 3.5	30.2	31.1 ± 2.2	33.8
5	0	0	3.7	2.5	3.5	0.2	108.9 ± 0.0	109.2	40.5 ± 0.0	41.2	63.1 ± 0.0	64.6
6	0	0	3.7	2.5	3.5	0.2	122.1 ± 0.0	109.2	35.9 ± 0.0	41.2	75.0 ± 0.0	64.6
7	0	0	3.7	2.5	3.5	0.2	100.7 ± 0.0	109.2	42.0 ± 0.0	41.2	69.0 ± 0.0	64.6
8	0	0	3.7	2.5	3.5	0.2	120.0 ± 0.0	109.2	41.1 ± 0.0	41.2	64.9 ± 0.0	64.6
9	+1	0	7.4	2.5	3.5	0.2	114.4 ± 13.6	120.2	74.2 ± 2.1	71.5	64.0 ± 3.4	74.7
10	-1	+1	0	5.0	7.0	0.4	62.2 ± 2.6	62.4	47.1 ± 2.8	47.9	29.1 ± 2.5	27.5
11	0	+1	3.7	5.0	7.0	0.4	125.3 ± 0.8	123.7	54.1 ± 0.2	53.5	44.9 ± 2.9	51.9
12	+1	+1	7.4	5.0	7.0	0.4	140.2 ± 8.0	140.7	78.8 ± 0.5	78.5	61.1 ± 1.9	55.6

*The cultivations were performed in triplicate, with the exception of cultivation at condition (0,0) performed in quadruplicate; SD = standard deviation.

## Results and discussion

### Individual effect of diamines and precursors on cephamycin C production

For this study, two concentrations for each diamine were defined based on literature data obtained for other beta-lactam antibiotic producing microorganisms [32,33,35,42]. Cephamycin C biosynthesis precursors lysine and alpha-aminoadipic acid were tested at several concentrations in order to define ranges of adequate values for the experimental designs.

**Figure 1:**
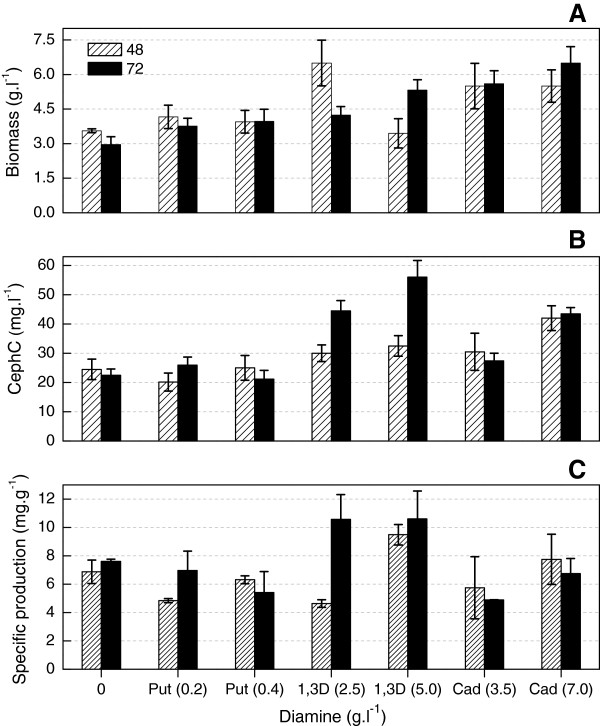
**Effect of biomass and cephamycin C with different diamines.** Biomass **(A)**, cephamycin C concentration (CephC) **(B)**, and specific production **(C)** obtained in shake-flasks cultivations of basal medium with no antibiotic-production enhancing compound (control condition) and with putrescine (Put), 1,3-diaminopropane (1,3D), and cadaverine (Cad), at two concentration values (in parentheses); the cultures were performed in triplicate.

Cephamycin C production and cell growth obtained at 48 h and 72 h cultivations in basal medium without additives and supplemented with putrescine, 1,3-diaminopropane, and cadaverine are shown in Figure 1. Leitão et al. [32] found that all three diamines promoted cephamycin C production by *N. lactamdurans*, albeit at different levels. The largest increase was observed in culture media containing 2.5 or 5.0 g l^-1^ of 1,3-diaminopropane. In this study, this diamine also produced a similar effect: a 100% increase in volumetric production was observed after the addition of 5.0 g l^-1^ of the compound as compared to that of the culture medium with no additive. Also, the addition of 1,3-diaminopropane alone promoted higher specific production than that obtained at the control condition (Figure 1C). Similarly, Martín et al. [42] observed that adding 5.0 mM (0.37 g l^-1^) or 10 mM (0.74 g l^-1^) of 1,3-diaminopropane enhanced *Penicillium chrysogenum* beta-lactam antibiotic production by approximately 100%. It is likely that one of the effects of 1,3-diaminopropane is to maintain high mRNA transcript levels during the production phase [43].

In the present work, putrescine did not affect antibiotic production by *S. clavuligerus* (Figure 1B), as Martín et al. [42,43] observed with *P. chrysogenum*. However, Leitão et al. [32] observed positive effects on cephamycin C production with *N. lactamdurans* when 0.20 g l^-1^ of putrescine was added.

With regard to cadaverine, volumetric production almost doubled by adding 7.0 g l^-1^ of this diamine (Figure 1B). However, specific production was not higher than that obtained in media without additives (Figure 1C). For cultivations with *N. lactamdurans*, a threefold increase was obtained using 5.0 g l^-1^ of this diamine [32].

In general, the increase in biomass observed at the end of cultivations (Figure 1A) suggests that these diamines acted as sources of carbon and energy (C) and/or nitrogen (N), thereby supplementing the basal medium sources (starch and PROFLO®).

**Figure 2:**
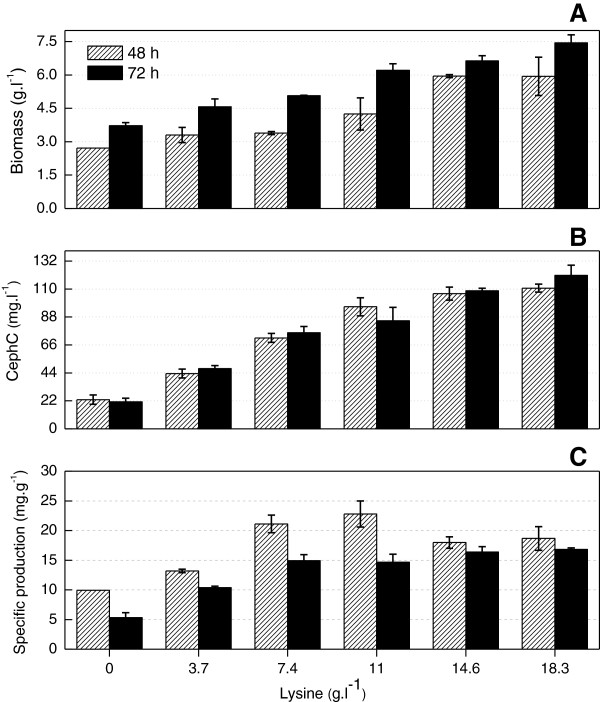
**Effect of biomass and cephamycin C with lysine.** Biomass **(A)**, cephamycin C concentration (CephC) **(B)**, and specific production **(C)** obtained from batch cultivations in shaken-flasks of basal medium with no antibiotic-production enhancing compound (control condition) and with lysine (Lys) at different concentration values; the cultures were performed in triplicate.

**Figure 3:**
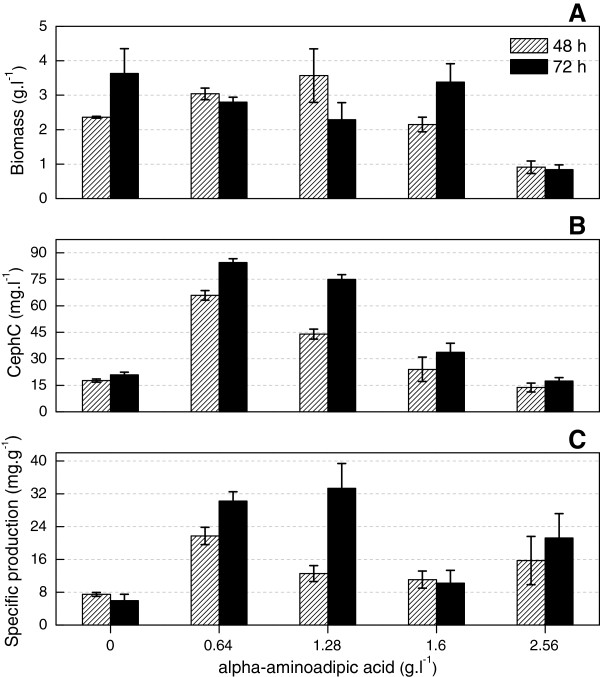
**Effect of biomass and cephamycin C with alpha-aminoadipic acid.** Biomass **(A)**, cephamycin C concentration (CephC) **(B)**, and specific production **(C)** obtained from batch cultivations in shaken-flasks of basal medium with no antibiotic-production enhancing compound (control condition) and with alpha-aminoadipic acid (AAA) at different concentration values; the cultures were performed in triplicate.

Cephamycin C production was evaluated at several lysine and alpha-aminoadipic acid concentrations (Figures 2 and 3). Consistent with the literature, high concentrations of exogenous lysine strongly affected cephamycin C production [20,28]. After adding 14.6 g l^-1^ of this amino acid, biomass almost doubled (Figure 2A) and cephamycin C production increased about six fold (Figure 2B) as compared to data from the basal medium. However, residual concentration values of this amino acid at 14.6 g l^-1^ and 18.3 g l^-1^ of lysine were approximately 25% and 35%, respectively. This surplus was not observed at concentrations lower than 11 g l^-1^. Moreover, a fivefold global increase in antibiotic volumetric production was obtained between 0 and 11 g l^-1^ of lysine, whereas biomass increased only 1.5 times.

Adding up to 1.6 g l^-1^ of alpha-aminoadipic acid did not influence biomass formation, which was in the same order of magnitude as that in the basal medium with no additives. Adding 0.64 g l^-1^ of alpha-aminoadipic acid to the basal medium resulted in the largest increase in cephamycin C production, four times larger than that obtained with the basal medium. Alpha-aminoadipic acid concentrations higher than 0.64 g l^-1^ did not promote higher antibiotic volumetric production, in spite of the amino acid having been completely consumed. Henriksen et al. [44] reported that alpha-aminoadipic acid can be metabolized into 6-oxo-piperideine-2-carboxylic acid (OPC), which is secreted into the culture medium during penicillin production by *P. chrysogenum*. The authors suggested that OPC formation would divert alpha-aminoadipic acid from antibiotic synthesis and lead to lower levels of penicillin production. A similar phenomenon may have occurred in *S. clavuligerus*.

### Effect of lysine in conjunction with diamines or alpha-aminoadipic acid on cephamycin C production

The concentration of independent variables enabled the investigation of ranges in which, according to the literature and data from this study, it is possible to maximize cephamycin C production by *S. clavuligerus* or *N. lactamdurans *[16,20,21,31-34,42,43]. Based on results of cultivations using only lysine as additive (Figure 2), concentrations of amino acid ranging from 0 to 7.4 g l^-1^ were selected in order to minimize its effect on biomass production. With respect to alpha-aminoadipic acid, concentrations ranging from 0 to 0.64 g l^-1^ were selected due to superior cephamycin C volumetric production results obtained in this range (Figure 3).

As to lysine, the highest volumetric production of cephamycin C was observed at 48 hours, which varied little at 72 hours (Figure 2B). The highest volumetric production values for the basal medium with 1,3-diaminopropane or alpha-aminoadipic acid were observed at 72 hours. With respect to cadaverine and putrescine, the highest volumetric production values observed at 48 and 72 hours were almost the same. For this reason, cultivation time was standardized to 72 hours for the experimental designs and bioreactor processes.

**Figure 4:**
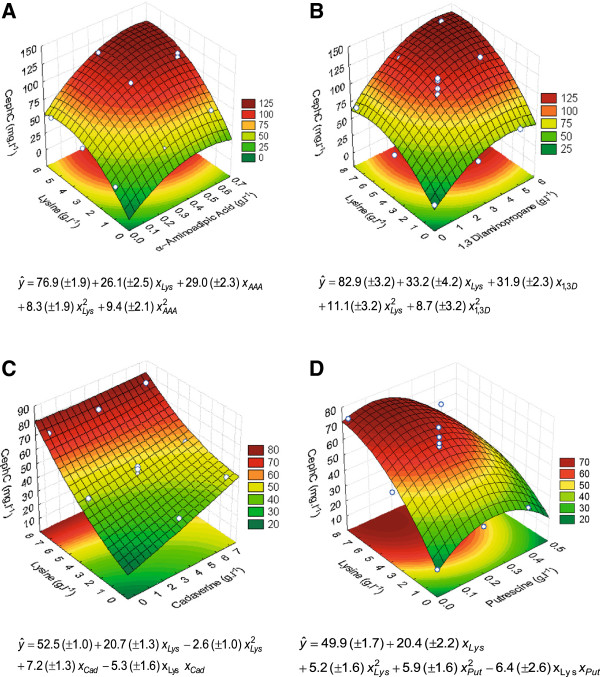
**Fitted response surfaces (at 90% confidence level) for cephamycin C concentration (CephC).** Batch cultivation (72-hour) in shaken-flasks in media containing: **(A)** lysine (Lys) and alpha-aminoadipic acid (AAA), **(B)** lysine (Lys) and 1,3-diaminopropane (1,3D), **(C)** lysine (Lys) and cadaverine (Cad), and **(D)** lysine (Lys) and putrescine (Put).

The chosen experimental design (CCF) and the concentration range employed for the compounds under investigation (independent variables), together with the use of response surface methodology for statistical treatment of the data obtained at 72 h cultivation, allowed for the adjustment of quadratic models to predict cephamycin C production at 90% confidence level. The generated response surfaces and their corresponding second-order polynomials are shown in Figure 4. Table 3 shows the analyses of variance (ANOVA) of the fitted models, including the F-test to verify the overall significance of each model, its associated probabilities p(F), and determination coefficient R^2^.

**Table 3:** Analyses of variance (ANOVA) for the quadratic models regressions at 90% confidence level

	**Lysine and alpha-aminoadipic acid (R**^**2**^ = 0.9543*)					**Lysine and 1,3-diaminopropane (R**^**2**^ = 0.9544*)				
**Source**	**SS**	**DF**	**MS**	**F**	**p**	**SS**	**DF**	**MS**	**F**	**p**
Model	13,068.14	5	2,613.63	25.06**	6.0 x 10^-4^	15,993.37	5	3198.67	25.10**	6.0 x 10^-4^
Residual	625.82	6	104.30			764.58	6	127.43		
Lack of fit	509.35	3	169.78	4.37	0.128	441.58	3	147.19	1.37	0.402
Pure error	116.47	3	38.82			323.00	3	107.67		
Total	13,693.96	11				16,757.95	11			
	**Lysine and cadaverine (R**^**2**^ = 0.9793*)					**Lysine and putrescine (R**^**2**^ = 0.9006*)				
**Source**	**SS**	**DF**	**MS**	**F**	**p**	**SS**	**DF**	**MS**	**F**	**p**
Model	3,080.16	5	616.03	56.77**	<10^-4^	3,650.07	5	730.01	10.87**	5.7 x 10^-3^
Residual	65.10	6	10.85			402.82	6	67.14		
Lack of fit	32.35	3	10.78	0.99	0.503	318.82	3	106.27	3.79	0.151
Pure error	32.75	3	10.92			84.00	3	28.00		
Total	3,145.26	11				4052.89	11			

*Coefficients of determination for the regressions at 90% confidence level; SS: Sum of Squares; DF: Degrees of Freedom; MS: Mean Square.

**F_(5,6) at 90% confidence level_ = 3.11.

Cephamycin C production was affected differently for lysine combined with the remaining four compounds. The resulting response surfaces of experimental designs using lysine and alpha-aminoadipic acid (Figure 4A) and lysine and 1,3-diaminopropane (Figure 4B) showed curves and parameters of the same order of magnitude, thereby providing comparable production values. The adjusted mathematical models provide the highest cephamycin C concentrations of approximately 126 and 140 mg l^-1^ when 0.6 g l^-1^ of alpha-aminoadipic acid and 5.3 g l^-1^ of lysine and 5.2 g l^-1^ of 1,3-diaminopropane and 7.0 g l^-1^ of lysine were added, respectively. In culture media containing just lysine, a production of about 120 mg l^-1^ was obtained, but only at high amino acid concentrations (14.6 g l^-1^) (Figure 2).

It should be remarked that alpha-aminoadipic acid has a strong impact on cephamycin C production even when added at concentrations nine times lower than those of 1,3-diaminopropane. This is probably due to its being a direct precursor of the beta-lactam antibiotic molecule [20,21,33]. On the other hand, 1,3-diaminopropane acts indirectly on beta-lactam antibiotic biosynthesis at the genetic and transcriptional levels [32,43]. Leitão et al. [32] showed that this diamine increases the concentration of lysine-6-aminotransferase and P6C dehydrogenase, which are enzymes responsible for alpha-aminoadipic acid formation. This complex mechanism may support the need for adding larger amounts of 1,3-diaminopropane to produce the same effect as that obtained with alpha-aminoadipic acid at lower concentrations, which is in line with the results obtained in this study. These data and those found in the literature clearly demonstrate, albeit through different methods, that lysine conversion to alpha-aminoadipic acid is a limiting step to cephamycin C biosynthesis. For this reason, adding alpha-aminoadipic acid or 1,3-diaminopropane, though at different concentration levels, was equally effective to overcoming this bottleneck.

Fitted response surfaces for cultivations in culture media containing lysine combined with cadaverine indicate that this diamine only exerts influence on antibiotic production when lysine is added at low concentrations. When the amino acid concentration was increased, the effect of adding diamine gradually waned. It has been suggested that intracellular accumulation of cadaverine may regulate the lysine catabolic pathway through a feedback control mechanism. In this manner, the lysine that would be decarboxylated to form cadaverine is spared, thus increasing lysine supply for cephamycin biosynthesis via the alpha-aminoadipate pathway. The fitted model shows that this behavior only happens at low lysine concentrations. At higher concentrations, lysine would supply both cadaverine and alpha-aminoadipic acid pathways, thereby decreasing the influence of cadaverine. For this reason, as predicted by the model, there is little antibiotic variation (73–77 mg l^-1^ of cephamycin C) at the highest lysine concentration (7.4 g l^-1^) within the entire cadaverine concentration range under investigation. This is due to the fact that the linear effect of lysine is about thrice stronger than that of this diamine.

With respect to lysine combined with putrescine, adding 0.20 g l^-1^ of this diamine to media containing 3.7 g l^-1^ of amino acid increased production by approximately 40% as compared to that obtained with medium containing just lysine at the same concentration (Table 2). On the other hand, adding this diamine to media with higher lysine concentrations (7.4 g l^-1^) adversely affected production due to the negative effect stemming from the interaction between the compounds (Figure 4D). Thus, the highest production value predicted for 7.7 g l^-1^ of lysine combined with 0.13 g l^-1^ of putrescine is just 76 mg l^-1^. Similar volumetric production values were obtained with basal culture media containing 7.4 g l^-1^ of lysine as additive (Figure 2). Martín et al. [43] observed that supplementation with putrescine provided much lower mRNA levels than those obtained with 1,3-diaminopropane in *P. chrysogenum* cultures. Despite structural similarity between 1,3-diaminopropane and putrescine, these authors suggest that the positive effect obtained with diamines is probably attributable to the three-carbon structure of diamines. On the other hand, Leitão et al. [32] observed an approximately threefold increase when 0.2 g l^-1^ of putrescine was added to *N. lactamdurans* cultures.

**Figure 5:**
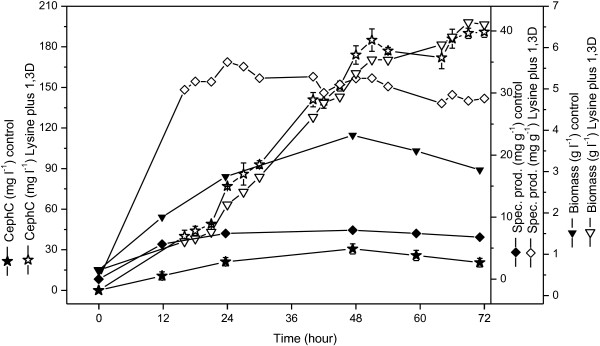
**Batch cultivation in agitated and aerated bench-bioreactor for lysine combined with 1,3-diaminopropane.** Cephamycin C concentration (CephC), specific production, and biomass; basal medium containing cephamycin C production-enhancing compounds at their optimal values (in parentheses), lysine (7.0 g l^-1^) and 1,3-diaminopropane (5.2 g l^-1^) (open symbols); control condition: basal medium without additives (solid symbols).

**Figure 6:**
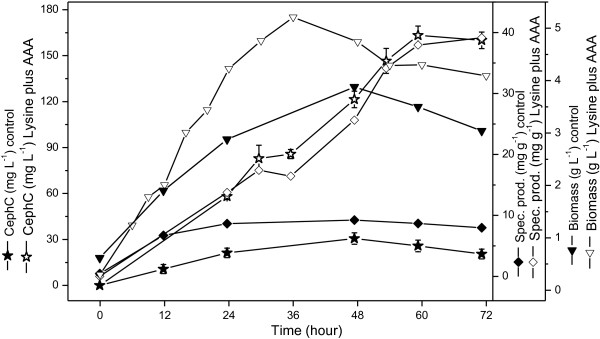
**Batch cultivation in agitated and aerated bench-bioreactor for lysine combined with alpha-aminoadipic acid.** Cephamycin C concentration (CephC), specific production, and biomass; basal medium containing cephamycin C production-enhancing compounds at their optimal values (in parentheses), lysine (5.3 g.l^-1^) and alpha-aminoadipic acid (0.6 g.l^-1^) (open symbols); control condition: basal medium without additives (solid symbols).

Figures 5 and 6 show the results of two cultivations in bioreactor using 7.0 g l^-1^ of lysine combined with 5.2 g l^-1^ of 1,3-diaminopropane and 5.3 g l^-1^of lysine combined with 0.64 g l^-1^ of alpha-aminoadipic acid. These concentrations, predicted by the models as optimal production conditions, resulted in 190 mg l^-1^ and 160 mg l^-1^ of cephamycin C for lysine combined with 1,3-diaminopropane and lysine combined with alpha-aminoadipic acid, respectively.

When compared to top values predicted by the mathematical models, these results represent increases of approximately 35% for lysine combined with 1,3-diaminopropane and approximately 27% for lysine combined with alpha-aminoadipic acid. While diamine supplementation favored cell growth, because it can act as an additional source of C and N, alpha-aminoadipic acid did not affect biomass production. Thus, the specific production at the end of cultivation with lysine combined with alpha-aminoadipic acid was approximately 30% higher as compared to that of lysine combined with 1,3-diaminopropane, reaching values of up to 40 mg l^-1^ and 30 mg l^-1^, respectively. Results obtained in bioreactor employing medium without additives (control condition) are also shown in Figures 5 and 6.

## Conclusions

It has been known for a long time that adding lysine enhances cephamycin C production. However, its use as the sole enhancer does not take full advantage of the antibiotic productivity of *S. clavuligerus*. In this study, an experimental design method (CCF) and Response Surface Methodology are successfully employed to adjust mathematical models to describe the effects of lysine combined with cadaverine, putrescine, 1,3-diaminopropane or alpha-aminoadipic acid on cephamycin C production by *S. clavuligerus*. Moreover, the interactions observed and validated by the fitted models are shown to be compatible to biochemical data already established in the literature about the pathway of beta-lactam antibiotics in *S. clavuligerus*. This study demonstrates that different combinations of lysine with other compounds promote significant variations in antibiotic production, with emphasis on the benefits obtained from using lysine combined with alpha-aminoadipic acid or 1,3-diaminopropane. These combinations increased cephamycin C production by more than 100% as compared to that with culture media containing just lysine as additive at the same concentrations. This positive effect may be attributed to alpha-aminoadipic acid or 1,3-diaminopropane in conjunction with lysine acting to overcome the bottleneck caused by lysine conversion to alpha-aminoadipic acid, albeit via different mechanisms. In the case of lysine combined with cadaverine, there was a positive effect on cephamycin C production by the diamine, especially when lysine was added at low concentrations. Cadaverine acted by decreasing lysine catabolism. However, as the amino acid concentration increased, the diamine effect waned, as the model clearly indicates. On the other hand, the highest volumetric production obtained with lysine combined with putrescine was approximately twice lower than that obtained with lysine combined with alpha-aminoadipic acid or 1,3-diaminopropane.

## Competing interests

All the authors of the submitted work (CA, AP, and MLGC) declare that there has been no financial relationship or support from any company in the past five years. We declare too that there are no competing interests, whether political, personal, religious, ideological, academic, intellectual or commercial, or any other activities influencing the submitted work.

## Authors’ contributions

CA carried out the assays with the diamines (experimental designs and fermentation in bioreactor), and was responsible for the agar bioassays and handling, storage, and maintenance of the microorganisms (*Streptomyces clavuligerus* ATCC 27064 and *Escherichia coli* ESS 2235). AP carried out the assays with alpha-aminoadipic acid (experimental designs and fermentation in bioreactor), and was responsible for the analyses in high-performance liquid chromatography (amino acids, C and N sources, antibiotics). MLGC designed and coordinated the study and performed its statistical analysis. All authors collaborated on the text, interpreting and discussing the results, and approved the final manuscript.

## Authors’ information

CA – biologist; PhD student in Biotechnology, Institute of Chemistry, Araraquara campus, Department of Biochemical and Technological Chemistry, Laboratory of Bioprocesses; AP – biochemist and pharmacist, PhD student in Biotechnology, Institute of Chemistry, Araraquara campus, Department of Biochemical and Technological Chemistry, Laboratory of Bioprocesses; MLGC – chemical engineer and PhD in Chemical Engineering and expertise in industrial microbiology and fermentation for almost 25 years, assistant Professor at UNESP (São Paulo State University) Institute of Chemistry, Araraquara campus, Department of Biochemical and Technological Chemistry, Laboratory of Bioprocesses.
